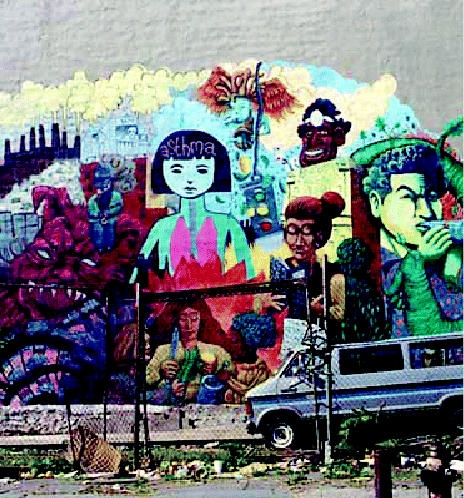# Street Science: Community Knowledge and Environmental Health Justice

**Published:** 2005-08

**Authors:** Julie Sze

**Affiliations:** Julie Sze is an assistant professor in American Studies at the University of California, Davis. Her research examines race and urban environmentalism, community-based planning and environmental health research. Her forthcoming book from MIT Press looks at environmental justice activism in New York City, asthma politics, and changes in garbage and energy resulting from privatization and deregulation.

Jason Corburn’s *Street Science: Community Knowledge and Environmental Health Justice* is an important addition to the literature on the science and politics of environmental health decision making. In clear prose, Corburn provides a “descriptive, analytic, and prescriptive understanding of local environmental-health knowledge” through what he calls “street science” (p. 217). Street science is a framework that joins local insights with professional scientific techniques, with concurrent goals: to improve scientific inquiry and environmental health policy and decision making.

At the heart of *Street Science* are four case studies from Greenpoint/ Williamsburg, in New York City, where diverse racial and ethnic, low-income populations practice what Corburn calls “science on the streets of Brooklyn.” These studies were centered on complex environmental health issues: subsistence fishing risks, asthma, childhood lead poisoning, and small sources of air pollution. Some of the larger issues addressed through these particular studies include the limits of traditional risk assessment and the politics of mapping health and environment risk. Through these studies, Corburn provides a theoretical model for understanding key characteristics of what he calls “local knowledge,” its paradoxes, and contributions to environmental health policy. Street science, at its best, identifies hazards and highlights research questions that professionals may ignore, provides hard-to-gather exposure data, involves difficult-to-reach populations, and expands possibilities for interventions, resulting in “improved science and democracy.” One of the strengths of this book is that it succeeds where most studies of local knowledge fail, “scaling up” and providing generalizations about the nature of local knowledge, how it is acquired, the typical problems that occur when local and scientific knowledge conflict and why.

Drawing from social science, particularly science and technology studies, Corburn explicitly calls for environmental and public health researchers, policy makers, and urban planners to become “reflective practitioners.” At the same time, he is careful to reject the idea that street science is a panacea. It does not devalue, but rather revalues science. He is not calling for a populism where the “community” replaces “experts,” but for a better understanding of how knowledge “co-produced” among local and professional constituencies can lead to better health, science, and politics.

The greatest strength of the book is in the details about the particular interventions that street science made in these four examples. One of the stronger cases was in the story about subsistence fishing. Local residents added to a U.S. Environmental Protection Agency (EPA) Air Toxics Modeling and Cumulative Exposure project by contributing local knowledge to the dietary exposure assessment. The U.S. EPA had no idea that local residents consumed contaminated fish from the East River, but as a result of community challenges to the U.S. EPA’s risk assessment models, the agency was able to conduct angler surveys and to more accurately represent the real-life exposures that local residents faced. Local knowledge was culturally sensitive, linked with the environmental justice movement, successfully used intermediaries, and was low-cost enough to be incorporated successfully into the U.S. EPA’s practices. Corburn does not claim that each example of street science is successful or equivalent with one another. But even these failures and limits are instructive. For policy makers and health researchers who face hostile communities, his accounts of conflictive public meetings in Greenpoint/ Williamsburg offer a good guide to “what goes wrong and why.”

Agencies such as the National Institute of Environmental Health Sciences are increasingly recognizing community-based research and environmental justice concerns [exemplified, for example, by “Advancing Environmental Justice through Community-Based Participatory Research,” Environ Health Perspect 110(suppl 2)]. At the same time, more focus and funding is being channeled into investigating and eliminating health disparities. Corburn’s *Street Science* is an essential and critical investigation into the science and politics of local knowledge and environmental health justice at this crucial juncture.

## Figures and Tables

**Figure:**